# Radiologic Assessment of Periprostatic Fat as an Indicator of Prostate Cancer Risk on Multiparametric MRI

**DOI:** 10.3390/bioengineering12080831

**Published:** 2025-07-31

**Authors:** Roxana Iacob, Emil Radu Iacob, Emil Robert Stoicescu, Diana Manolescu, Laura Andreea Ghenciu, Radu Căprariu, Amalia Constantinescu, Iulia Ciobanu, Răzvan Bardan, Alin Cumpănaș

**Affiliations:** 1 Doctoral School, ‘Victor Babes’ University of Medicine and Pharmacy Timisoara, Eftimie Murgu Square No. 2, 300041 Timisoara, Romania; roxana.iacob@umft.ro (R.I.); amalia.constantinescu@umft.ro (A.C.); 2 Department of Anatomy and Embriology, ‘Victor Babes’ University of Medicine and Pharmacy Timisoara, Eftimie Murgu Square No. 2, 300041 Timisoara, Romania; ciobanu.iulia@umft.ro; 3 Research Center for Medical Communication, ‘Victor Babes’ University of Medicine and Pharmacy Timisoara, Eftimie Murgu Square No. 2, 300041 Timisoara, Romania; 4 Field of Applied Engineering Sciences, Specialization Statistical Methods and Techniques in Health and Clinical Research, Faculty of Mechanics, ‘Politehnica’ University Timisoara, Mihai Viteazul Boulevard No. 1, 300222 Timisoara, Romania; 5 Department of Pediatric Surgery, ‘Victor Babes’ University of Medicine and Pharmacy Timisoara, 300041 Timisoara, Romania; 6 Department of Radiology and Medical Imaging, ‘Victor Babes’ University of Medicine and Pharmacy Timisoara, Eftimie Murgu Square No. 2, 300041 Timisoara, Romania; dmanolescu@umft.ro (D.M.); radu.caprariu@umft.ro (R.C.); 7 Research Center for Pharmaco-Toxicological Evaluations, ‘Victor Babes’ University of Medicine and Pharmacy Timisoara, Eftimie Murgu Square No. 2, 300041 Timisoara, Romania; 8 Department of Functional Sciences, ‘Victor Babes’ University of Medicine and Pharmacy Timisoara, Eftimie Murgu Square No. 2, 300041 Timisoara, Romania; bolintineanu.laura@umft.ro; 9 Department of Urology, ‘Victor Babes’ University of Medicine and Pharmacy Timisoara, Eftimie Murgu Square No. 2, 300041 Timisoara, Romania; bardan.razvan@umft.ro (R.B.); cumpanas.alin@umft.ro (A.C.)

**Keywords:** prostate cancer, prostate bpMRI, periprostatic adipose tissue, subcutaneous fat thickness, prostate cancer imaging, non-invasive cancer markers, tumor microenvironment

## Abstract

Prostate cancer remains one of the most prevalent malignancies among men, and emerging evidence proposed a potential role for periprostatic adipose tissue (PPAT) in tumor progression. However, its relationship with imaging-based risk stratification systems such as PI-RADS remains uncertain. This retrospective observational study aimed to evaluate whether periprostatic and subcutaneous fat thickness are associated with PI-RADS scores or PSA levels in biopsy-naïve patients. We retrospectively reviewed 104 prostate MRI scans performed between January 2020 and January 2024. Fat thickness was measured on axial T2-weighted images, and statistical analyses were conducted using Spearman’s correlation and multiple linear regression. In addition to linear measurements, we also assessed periprostatic fat volume and posterior fat thickness derived from imaging data. No significant correlations were observed between fat thickness (either periprostatic or subcutaneous) and PI-RADS score or PSA values. Similarly, periprostatic fat volume showed only a weak, non-significant correlation with PI-RADS, while posterior fat thickness demonstrated a weak but statistically significant positive association. Additionally, subgroup comparisons between low-risk (PI-RADS < 4) and high-risk (PI-RADS ≥ 4) patients showed no meaningful differences in fat measurements. These findings suggest that simple linear fat thickness measurements may not enhance imaging-based risk assessment in prostate cancer, though regional and volumetric assessments could offer modest added value.

## 1. Introduction

The prostate is a small, walnut-sized gland situated in the pelvis, positioned below the bladder, in front of the rectum, and surrounding the male urethra [1,2]. Its primary role is to produce prostatic fluid, a key component of semen that nourishes and protects sperm [3]. Common prostate conditions include BPH, prostatitis, and prostate cancer, which varies from slow-growing to aggressive [1].

Prostate cancer is among the most frequent malignancies in men, with its incidence rising significantly after the age of 50 [4]. Often asymptomatic early on, prostate cancer may later cause urinary issues, blood in urine or semen, pelvic pain, or bone pain from metastasis [4,5]. Prostate cancer is diagnosed through a combination of clinical evaluation and diagnostic tests [6]. The primary screening methods include prostate-specific antigen (PSA) blood testing and digital rectal examination (DRE) [6]. PSA levels under 2.5 ng/mL (under 60) or 4.0 ng/mL (over 60) are considered normal. Levels between 4 and 10 ng/mL may indicate BPH or cancer, while those above 10 ng/mL suggest higher cancer risk [7]. If abnormalities are detected, further evaluation with multiparametric MRI (mpMRI) can help identify suspicious lesions [8]. Lesions on mpMRI are assessed using PI-RADS, a standardized score estimating prostate cancer risk and guiding biopsy decisions [9]. In recent years, biparametric MRI (bpMRI) gained attention as a radiation-free and faster alternative mpMRI for prostate cancer detection [10,11]. bpMRI combines T2-weighted and diffusion-weighted imaging without contrast, reducing scan time and costs while still offering high diagnostic accuracy for clinically significant prostate cancer [10,12,13]. Its role in prostate cancer screening is under study, with evidence supporting its use, especially in low-resource settings or when contrast is contraindicated [10,14]. Early detection and timely management of prostate cancer are essential for achieving the best possible outcomes [15]. When detected early, prostate cancer is often confined to the gland and treatable with curative intent, improving survival and quality of life [15,16,17]. Delays in diagnosis or treatment can allow the disease to progress, making it more difficult to manage and reducing the chances of a favorable prognosis [16,18]. Because of this, researchers are actively identifying key signs and reliable markers of early-stage prostate cancer, aiming to improve screening and enable faster, more accurate diagnosis [19,20]. PPAT, which surrounds the prostate, secretes inflammatory cytokines and adipokines that may promote tumor growth and progression. Increased PPAT volume has been linked to higher PSA levels, elevated Gleason scores, and more aggressive disease [21]. As a result, PPAT is being investigated as a potential imaging biomarker for prostate cancer risk assessment and prognosis [22].

This study aims to evaluate the correlation between PPAT and PI-RADS scores to determine whether PPAT can serve as a reliable imaging biomarker for prostate cancer assessment. Specifically, it seeks to identify from which PI-RADS score this correlation becomes significant or if PPAT is more relevant only in biopsy-confirmed cases, as suggested by previous studies. By analyzing PPAT characteristics—including total PPAT volume and posterior PPAT thickness—in relation to PI-RADS classification, this research could provide insights into its potential role in early risk stratification and diagnosis. PPAT is best assessed using T1-weighted and T2-weighted MRI sequences, which clearly define fat distribution [10]. Some studies also explore DWI and MR spectroscopy for metabolic insights, but most of the related papers suggest that T1W and T2W remain the most used for PPAT evaluation [11]. Establishing a reliable association between PPAT and PI-RADS could support the future use of simplified imaging protocols such as bpMRI, improving non-invasive risk assessment and helping guide biopsy decisions. To reflect real-life clinical scenarios where MRI guides the need for biopsy, this study focused solely on biopsy-naive patients and evaluated MRI-derived parameters, including PPAT, in a clean, untreated population.

Given the objective of identifying early and non-invasive methods for prostate cancer risk stratification, this study used the PI-RADS imaging score as the main outcome marker. Although PI-RADS is not a histological endpoint, it is widely applied in clinical practice to guide biopsy decisions. Therefore, analyzing the relationship between PPAT characteristics and PI-RADS scores in biopsy-naïve patients may provide valuable insights into the potential role of imaging biomarkers for estimating cancer risk without relying on invasive procedures.

## 2. Materials and Methods

### 2.1. Study Design and Ethical Approval

Prostate mpMRIs performed at ‘Dr. Victor Babes’ Hospital of Infectious Diseases and Pneumophthisiology Timisoara, Romania between January 2020 and January 2024 were reviewed. Exclusions included scans of men under 40 years old, incomplete studies, cases with a history of prostate neoplasia or prior interventions on the prostate or periprostatic tissue, as well as patients with previously confirmed positive prostate biopsies; only biopsy-naive patients were included in the final analysis. After applying these criteria, 104 prostate mpMRI studies were deemed eligible for analysis. The study was conducted and approved by the Ethics Committee of ‘Dr. Victor Babes’ Hospital of Infectious Diseases and Pneumophtisiology Timisoara, Romania (approval number 8521/25 September 2024).

Although a subset of patients, the ones with PI-RADS 4 and 5 lesions, were recommended for targeted prostate biopsy, only a portion consented to undergo the minimally invasive procedure within our institution. Others either refused the intervention altogether or were referred to different medical centers for further evaluation. Consequently, histopathological confirmation was not uniformly available for the cohort. In order to avoid introducing bias due to the limited number of biopsy-confirmed cases, we opted to conduct a purely imaging-based statistical analysis.

### 2.2. Imaging Protocol and Measurement Methodology

**Figure 1:**
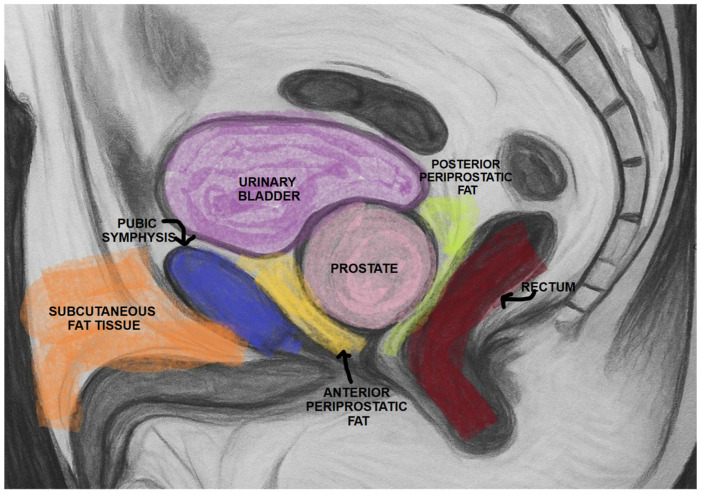
Schematic sagittal section of the male pelvis on MRI.

For all patients, PPAT thickness (PPATT), posterior periprostatic adipose tissue thickness (PPPATT), and subcutaneous adipose tissue thickness (SATT) were measured on T2-weighted MRI images by a radiologist with experience in prostate MRI using RadiAnt DICOM Viewer 2025.2 (Medixant: Poznań, Poland, https://www.radiantviewer.com/). T2W images not only provide clear visualization of fat distribution, but also represent the most appropriate sequence for such measurements and are one of the core components of biparametric MRI protocols. In addition to these linear measurements, volumetric analysis of periprostatic adipose tissue was also performed, providing a more comprehensive evaluation of fat distribution. This dual approach allowed for both simplified morphometric assessment and a more nuanced spatial quantification of adiposity. The image below represents a schematic sagittal MRI view of the male pelvic region (Figure 1).

**Figure 2:**
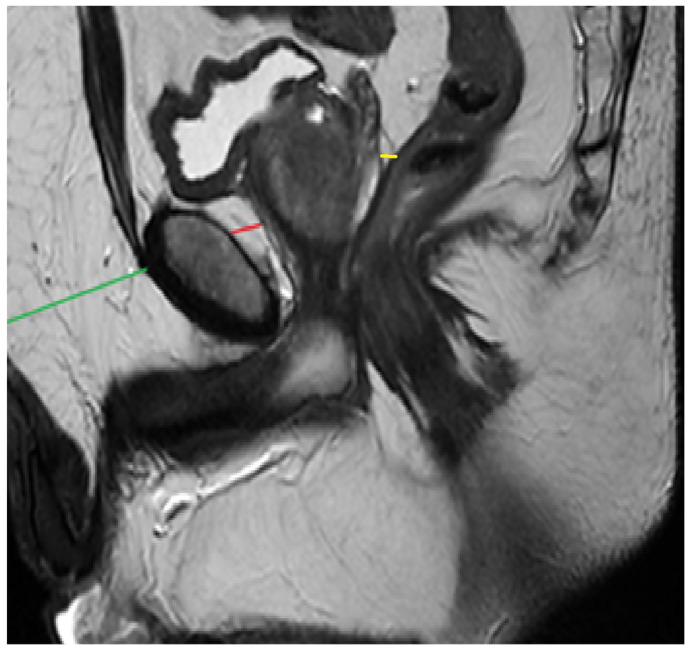
Measurement of PPAT (red line), posterior PPAT (yellow line), and SATT (green line) on a midsagittal view, T2 sequence, on a prostate MRI categorized as PI-RADS 2, showing a prostate containing a well-defined prostatoc cyst.

**Figure 3:**
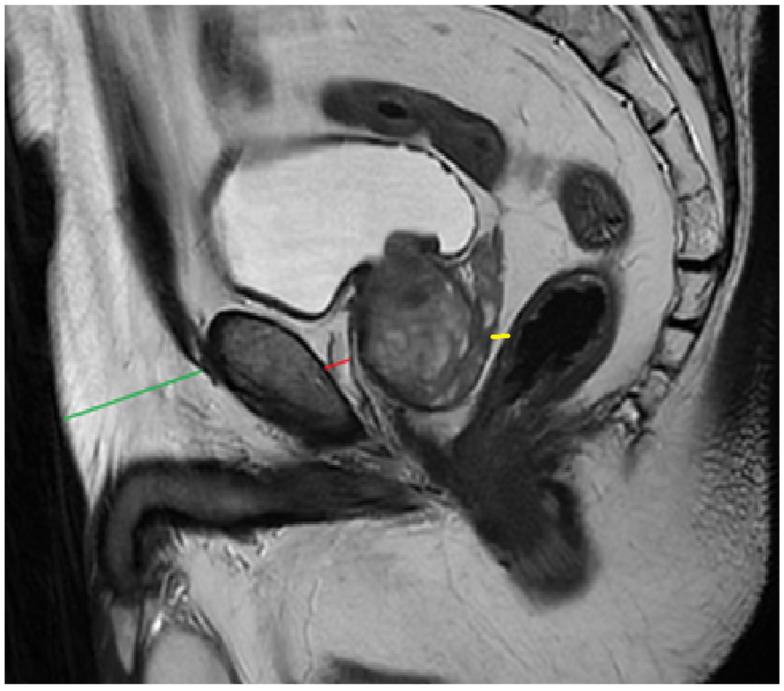
Measurement of PPAT (red line), posterior PPAT (yellow line), and SATT (green line) on a midsagittal view, T2 sequence, on a prostate MRI categorized as PI-RADS 4, showing a heterogeneous prostate gland that indents and protrudes into the urinary bladder.

PPATT was measured as the shortest vertical distance from the pubic symphysis to the anterior surface of the prostate on midsagittal images; SATT was measured on the same slice as PPATT, defined as the shortest vertical distance from the pubic symphysis to the overlying skin, while PPPATT was measured as the shortest distance from the posterior surface of the prostate and anterior wall of the rectum. The images below represent the visual explanation of these measurements (Figure 2 and Figure 3).

Periprostatic fat volume was quantified using 3D Slicer version 5.8.1, an open-source medical imaging software. Semi-automatic segmentation and analysis tools were employed to delineate the periprostatic fat and calculate its volume from the MRI data [12].

PI-RADS scores were assigned to each mpMRI examination based on the standardized PI-RADS version 2.1 criteria. Image interpretation was performed independently by two radiologists, each with over five years of experience in prostate MRI evaluation. In cases of discrepancy, consensus was reached through joint review to ensure consistency and accuracy in lesion assessment.

**Figure 4:**
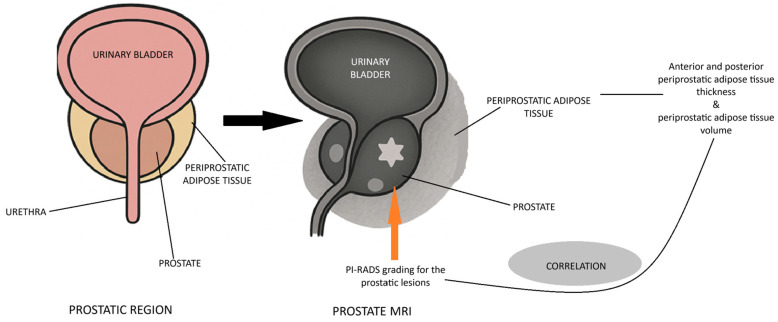
Rationale for the study.

The rationale for the study is presented in the figure below (Figure 4).

### 2.3. Statistical Methods and Analytical Approach

**Figure 5:**
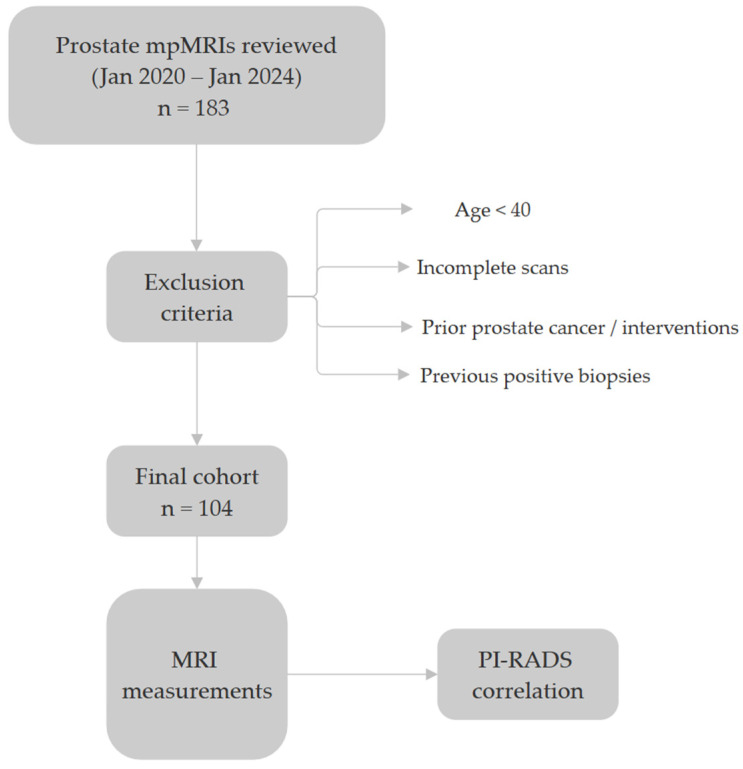
The methodology of the study.

Statistical analysis was primarily performed using MedCalc^®^ Statistical Software version 23.0. (MedCalc Software Ltd., Ostend, Belgium). The Shapiro–Wilk test was applied to assess the normality of continuous variables, revealing a non-Gaussian distribution for most of them. Accordingly, non-parametric statistical methods were used throughout the analysis. Spearman’s rank correlation coefficient (Spearman’s rho) was employed to evaluate associations between PPATT, SATT, PI-RADS scores, and PSA levels. The Mann–Whitney U test was used to compare fat thickness between low (PI-RADS < 4) and high (PI-RADS ≥ 4) risk groups. A multiple linear regression model was constructed to assess the predictive value of PPATT and SATT on PI-RADS scores. Figure 5 below shows the simplified methodology of the study.

**Figure 6:**
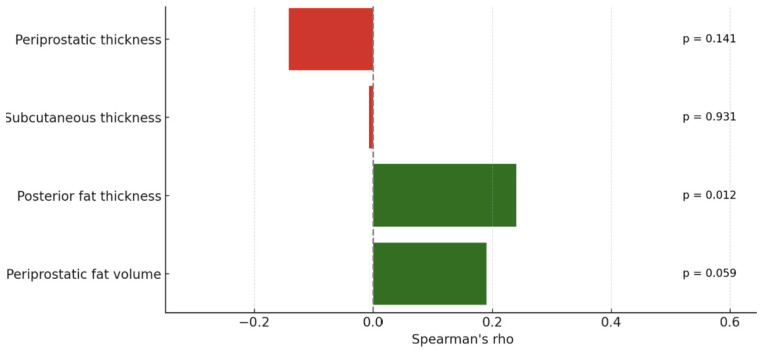
Correlation of adipose metrics with PI-RADS score. Green bars represent positive correlations, while red bars indicate negative correlations between each fat measurement and the studied variable.

**Figure 7:**
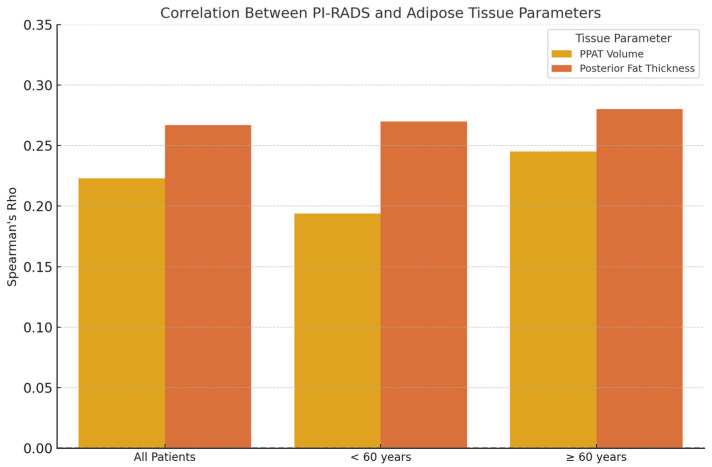
Spearman’s correlation coefficients (ρ) between PI-RADS score and adipose tissue parameters, stratified by age group. A modest positive correlation is observed between posterior fat thickness and PI-RADS in all age groups, while periprostatic fat volume shows a slightly stronger correlation in patients aged ≥ 60 years.

**Figure 8:**
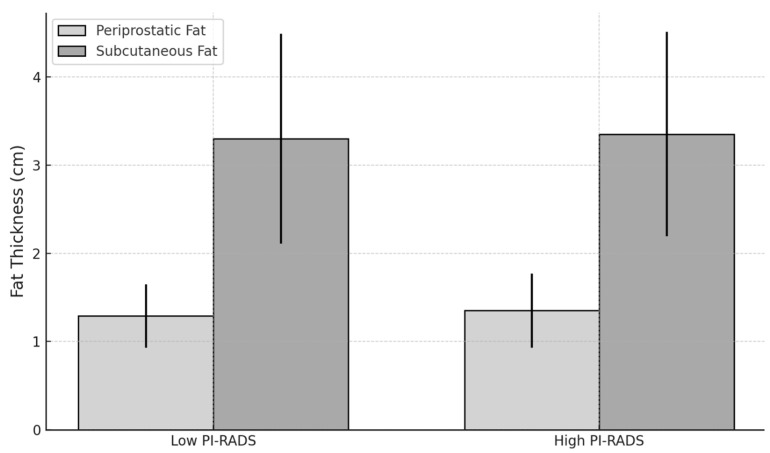
Chart comparing fat thickness between low- and high-PI-RADS groups.

To ensure reproducibility and increase transparency, all main statistical results were cross validated using Python (version 3.10). The “scipy.stats” package was used to replicate non-parametric testing and correlation analyses, while the statsmodels package was used for linear regression modeling. Figure 6, Figure 7 and Figure 8 were generated using Python’s “matplotlib” and “seaborn” libraries for high-quality data visualization. Statistical significance was set at *p* < 0.05 for all comparisons.

## 3. Results

### 3.1. Descriptive Statistics

A total of 104 patients were included (mean age 66.2 ± 10.97 years; range 40–87). Mean prostate volume on mpMRI was 45.8 ± 22.08 mL (range 9–145). The median PI-RADS score was 2 (IQR 2–4; range 1–5). Mean periprostatic fat thickness was 1.30 ± 0.37 cm (range 0.6–2.1) and mean subcutaneous fat thickness was 3.31 ± 1.18 cm (range 0.8–6.3). PSA levels had a median of 12.2 ng/mL (IQR 5.2–23.55; range 1.2–134.4). The demographic data of the 104 patients can be also found in the table below (Table 1).

### 3.2. Correlation Analysis

We analyzed the relationship between PI-RADS scores, PSA levels, and various adipose tissue measurements using Spearman’s correlation due to non-normal data distribution. No significant correlations were observed between PI-RADS scores and either periprostatic (ρ = −0.142, *p* = 0.141) or subcutaneous fat thickness (ρ = −0.007, *p* = 0.931). However, posterior fat thickness showed a weak but statistically significant positive correlation with PI-RADS (ρ = 0.24, *p* = 0.012), while periprostatic fat volume showed a similar trend that did not reach significance (ρ = 0.19, *p* = 0.059). PSA levels also showed weak, non-significant correlations with periprostatic (ρ = −0.162, *p* = 0.094) and subcutaneous fat thickness (ρ = −0.036, *p* = 0.718). The following figure shows the correlation of adipose metrics with PI-RADS score (Figure 6).

These results indicate no significant relationships between PI-RADS or PSA levels and either periprostatic or subcutaneous adipose tissue thickness in this cohort. While linear fat measurements appear to lack predictive value, regional (e.g., posterior) and volumetric assessments may offer modest correlations and could serve as more informative imaging markers.

### 3.3. Age-Stratified Correlation Analysis

To evaluate whether adipose tissue parameters differed by age group, we performed subgroup analyses for patients under 60 years (n = 26) and those aged 60 or above (n = 78). The strength and significance of correlations between PI-RADS scores and fat-related metrics varied across age categories.

For the entire cohort, PI-RADS showed a statistically significant, weak positive correlation with periprostatic fat volume (ρ = 0.223, *p* = 0.022) and posterior adipose tissue thickness (ρ = 0.267, *p* = 0.006), suggesting a general association between higher adiposity and increased PI-RADS scores.

In patients under 60 years, periprostatic fat volume demonstrated a moderate correlation with PI-RADS (ρ = 0.470, *p* = 0.0154), while posterior fat thickness did not reach statistical significance (ρ = 0.270, *p* = 0.180). Additionally, PSA levels were weakly but significantly correlated with PI-RADS (ρ = 0.408, *p* = 0.0381), and prostate volume showed a strong association (ρ = 0.707, *p* < 0.0001). Age also correlated moderately with PI-RADS in this subgroup (ρ = 0.496, *p* = 0.0099). No significant correlations were observed between PI-RADS and subcutaneous adipose thickness in younger patients.

In patients aged 60 and above, PSA levels showed a strong and highly significant correlation with PI-RADS scores (ρ = 0.671, *p* < 0.0001). Weak but statistically significant associations were also observed with posterior fat thickness (ρ = 0.280, *p* = 0.013), prostate volume (ρ = 0.299, *p* = 0.0078), and linear periprostatic fat thickness (ρ = 0.256, *p* = 0.0245). However, periprostatic fat volume was not significantly correlated with PI-RADS in this age group (ρ = 0.141, *p* = 0.2178). Interestingly, subcutaneous fat thickness showed a weak inverse correlation with PI-RADS (ρ = −0.226, *p* = 0.0467). Figure 7 shows the Spearman’s correlation coefficients between PI-RADS score and adipose tissue parameters, stratified by age group.

### 3.4. Multiple Regression Analysis

To further investigate the potential predictive value of adipose tissue thickness in explaining PI-RADS scores, we conducted a multiple linear regression analysis. The independent variables were periprostatic adipose tissue thickness and subcutaneous adipose tissue thickness, and the dependent variable was PI-RADS score.

The overall model was not statistically significant (F(2,100) = 0.987, *p* = 0.376), with an R-squared value of 0.019, indicating that only 1.9% of the variability in PI-RADS scores could be explained by periprostatic and subcutaneous adipose tissue thickness.

The coefficient for periprostatic adipose tissue thickness was −0.503 (*p* = 0.186), suggesting a weak, non-significant negative association with PI-RADS scores.

The coefficient for subcutaneous adipose tissue thickness was −0.020 (*p* = 0.865), indicating no significant relationship with PI-RADS scores.

### 3.5. Subgroup Analysis: Higher PI-RADS Scores

A subgroup analysis was performed by categorizing patients into high PI-RADS (≥4) and low PI-RADS (<4) groups to explore differences in adipose tissue thickness between these categories.

The mean periprostatic adipose tissue thickness in the high PI-RADS group was 1.35 cm (SD = 0.42), compared to 1.29 cm (SD = 0.36) in the low PI-RADS group. However, the difference was not statistically significant (*p* = 0.421, Mann–Whitney U test).

The mean subcutaneous adipose tissue thickness was 3.35 cm (SD = 1.16) in the high PI-RADS group and 3.30 cm (SD = 1.19) in the low PI-RADS group, with no significant difference (*p* = 0.903, Mann–Whitney U test).

Figure 8 below shows the comparison between fat thickness and low- and high-PI-RADS groups.

A summary of the most relevant correlations and statistical findings is presented in Table 2 below, offering a concise synthesis of the main results.

## 4. Discussion

Despite growing evidence that periprostatic adipose tissue (PPAT) may influence tumor progression through mechanisms such as inflammation, adipokine signaling, and hormonal regulation, our findings suggest that these biological effects may not yet be reflected in early imaging-based risk assessments. In our biopsy-naïve cohort, neither linear nor volumetric fat measurements showed strong associations with PI-RADS scores or PSA levels, indicating that basic morphometric analysis may have limited value in detecting subtle adiposity-related influences on lesion aggressiveness at this stage [13].

While linear fat measurements are easy to implement and widely used in clinical practice, our study also explored volumetric and regional assessments, which may better capture the biological impact of adiposity on local tumor behavior.

Previous studies reporting positive associations between PPATT and SATT and prostate cancer largely focused on patients with histologically confirmed malignancy, evaluating correlations with tumor aggressiveness, staging, or recurrence [14,15]. By contrast, our study involved a biopsy-naive population, where PI-RADS scores were used as indicators of suspicion rather than confirmed cancer. This distinction is important, as some lesions classified as PI-RADS 4 or 5 may have represented indolent disease or false positives, which may not be significantly influenced by local or systemic adiposity. Thus, direct comparison with earlier findings must be made with caution.

What distinguishes our study is its focus on a biopsy-naïve population, aiming to assess whether adipose tissue metrics can support non-invasive imaging-based risk stratification. Unlike prior research conducted on histologically confirmed cancer cases, our approach avoids selection bias and better reflects real-world scenarios where MRI precedes tissue diagnosis. To our knowledge, this is among the first studies to examine both linear and volumetric PPAT features in relation to PI-RADS using MRI, offering a novel perspective in prostate cancer risk assessment. Furthermore, by including an age-stratified analysis, we explored how adipose tissue parameters may differentially influence PI-RADS scores in younger versus older patients, highlighting possible age-related differences in the imaging expression of local adiposity and its association with lesion suspicion.

Additionally, inter-individual variability in fat distribution may not align with the early development of prostate cancer or with changes that are detectable through bpMRI, particularly in a relatively small and heterogeneous cohort. Since PI-RADS encompasses a wide range of lesions—including small or equivocal findings of uncertain clinical significance—any subtle associations with adiposity may have been diluted within this mixed cohort.

In our study, age-stratified analysis revealed notable differences in how adipose tissue metrics relate to PI-RADS scores. Among younger patients (under 60), periprostatic fat volume showed a clearer association with lesion suspicion, suggesting that early metabolic or inflammatory activity in the local periprostatic environment may influence how lesions are visualized with MRI. This could indicate that, in younger men, volumetric fat accumulation plays a more prominent role in the early phases of prostate cancer development or detection. Conversely, in patients aged 60 and above, localized fat measurements—particularly posterior and linear periprostatic thickness—were more closely associated with PI-RADS scores, while total fat volume appeared less informative. Subcutaneous fat thickness showed a weak inverse trend in this group, potentially reflecting differences between systemic and periprostatic fat behavior. These findings highlight the possibility that age-related anatomical and metabolic changes modulate the influence of adipose tissue on prostate cancer imaging, and that the relevance of various fat parameters may differ depending on patient age.

Volumetric assessment of periprostatic adipose tissue has been increasingly linked to features of tumor aggressiveness and a higher risk of biochemical recurrence, suggesting that this fat depot may actively contribute to prostate cancer progression. Increased PPAT volume observed with imaging has been associated with unfavorable pathological characteristics, while subcutaneous fat appears to have limited relevance in this context. These insights align with our findings, which show a weak positive correlation between PPAT volume and PI-RADS scores, particularly in younger patients. Additionally, posterior periprostatic fat thickness demonstrated a subtle association with PI-RADS scores in older individuals, indicating that regional fat distribution may influence lesion detectability in MRI. Therefore, while simple linear measurements may lack sufficient sensitivity for risk stratification in biopsy-naïve populations, volumetric evaluation of PPAT could offer greater predictive value in select clinical settings [16,18].

Recent studies emphasized that not all periprostatic adiposity metrics are equivalent in their clinical relevance. Notably, Liu et al. demonstrated that MRI-derived volumetric assessment of periprostatic fat—especially when normalized to prostate volume—outperformed linear measurements in predicting metastatic risk at diagnosis [10]. Likewise, Xiong et al. reported that posterior periprostatic fat thickness, combined with subcutaneous fat parameters, significantly improved MRI-based prediction models for prostate cancer detection [17]. These findings align with our results, which show stronger associations between PI-RADS scores and both posterior PPAT thickness and total PPAT volume, particularly when stratified by age. Together, these data support the value of regional and volumetric PPAT measurements as more informative imaging biomarkers than simple linear metrics. Future research could investigate whether specific features of periprostatic adipose tissue contribute to refining risk assessment in equivocal cases, particularly in lesions scored as PI-RADS 3, where the decision to proceed with biopsy remains clinically ambiguous. In this context, radiomics and AI-based approaches offer a logical and promising extension to current volumetric methods. By extracting advanced quantitative features from bpMRI—such as shape, texture, and spatial distribution of signal intensities—radiomics can identify subtle tissue patterns that are not detectable through conventional visual analysis. When integrated into machine learning models, these features may improve the accuracy of lesion classification and support decision-making in ambiguous cases, particularly for PI-RADS 3 lesions. Recent studies have shown that incorporating periprostatic fat metrics into AI-enhanced models improves diagnostic performance and enhances risk prediction at the time of prostate MRI, even in biopsy-naïve populations [19,20,23].

Building on these findings, a key direction for future work involves exploring advanced imaging analytics, particularly radiomics and AI-based models, for more robust risk stratification in prostate cancer. Radiomics can extract high-dimensional quantitative features from periprostatic and intratumoral regions, capturing microscopic heterogeneity that escapes visual detection. Recent studies have shown that radiomics-based models outperform traditional nomograms in predicting lymph node involvement [19], tumor aggressiveness [20], and even biochemical recurrence-free survival [23] after radical prostatectomy. Notably, radiomic features from periprostatic fat have shown comparable—and in some cases superior—prognostic value to intratumoral features, especially when integrated into radiomics-clinical nomograms. These approaches offer promising tools for improving lesion classification, especially in equivocal cases such as PI-RADS 3, where current guidelines offer limited clarity. Future AI-based models could assist in biopsy decision-making, reduce overtreatment, and personalize follow-up or adjuvant therapy strategies based on individualized risk. Moreover, integrating radiomic data with metabolic or inflammatory markers, and validating such models in large, prospective, multicenter cohorts, is essential to ensure robustness and generalizability.

Standardization of imaging protocols and measurement techniques for adipose tissue assessment is also essential to ensure consistency and comparability across studies. Even if periprostatic fat metrics do not directly impact initial diagnostic decisions, they may still offer value in tracking disease evolution, assessing treatment response, or identifying individuals at higher risk of progression. These possibilities support continued investigation into the role of adipose tissue as a complementary imaging biomarker in prostate cancer. Additionally, future research should consider adipose tissue parameters alongside other clinical variables—such as metabolic syndrome components, systemic inflammatory markers (e.g., CRP, IL-6), and genetic or epigenetic factors—to develop comprehensive, multifactorial risk prediction models. Such integrative approaches have the potential to enhance early prostate cancer detection, improve patient selection for biopsy or advanced imaging, and support more personalized diagnostic and therapeutic strategies.

Nonetheless, this study has several important limitations that must be acknowledged. First, the lack of histopathological confirmation across the entire cohort limits the strength of our conclusions. Since PI-RADS scores indicate suspicion rather than definitive malignancy, we cannot confirm whether the observed imaging findings correspond to clinically significant prostate cancer. Although some patients with PI-RADS 4 or 5 lesions underwent biopsy, others declined or were referred elsewhere, limiting our ability to validate the imaging–adiposity associations with histology. Second, the retrospective, single-center design may introduce selection bias and reduces generalizability. Our patient cohort reflects specific institutional workflows, and findings may not be applicable to other clinical settings or populations. Prospective multicenter validation would be essential to support broader implementation. Third, the absence of key clinical covariates, such as body mass index (BMI), metabolic syndrome components, and systemic inflammatory markers (e.g., CRP, IL-6), may have led to residual confounding. These unmeasured factors could influence both fat distribution and prostate cancer risk, complicating interpretation of imaging-based associations. Finally, the sample size, though sufficient for exploratory purposes, may have limited our power to detect subtle associations or to fully explore stratified analyses. Heterogeneity in lesion suspicion (especially in PI-RADS 3) could also have diluted potential trends.

Importantly, both the fat-related parameters evaluated in this study and the imaging classification system (PI-RADS) are fully compatible with label-free diagnostic protocols. PPAT is optimally visualized and measured on T2-weighted sequences, which do not require intravenous contrast agents. Moreover, prior studies demonstrated that bpMRI—based on T2-weighted and diffusion-weighted imaging—offers diagnostic accuracy comparable to mpMRI for clinically significant prostate cancer [24,25]. Therefore, this study supports the feasibility of developing non-invasive, contrast-free imaging approaches for risk stratification in biopsy-naïve patients, with potential applicability in resource-limited settings or in individuals with contraindications to contrast media.

Future studies should also aim to incorporate broader clinical variables and biopsy validation to enhance the generalizability and diagnostic relevance of imaging-based adiposity markers.

## 5. Conclusions

This study found no significant overall correlation between periprostatic or subcutaneous adipose tissue thickness and PI-RADS scores or PSA levels in biopsy-naive patients undergoing mpMRI. However, when assessing periprostatic fat volume and posterior fat thickness, weak positive correlations with PI-RADS were observed—particularly in age-stratified analysis, where fat volume was more relevant in younger patients and posterior fat thickness in older patients. These findings suggest that simple linear thickness may be insufficient, but regional and volumetric fat assessment could offer added value for risk stratification, especially in the context of bpMRI. Further research is warranted to explore whether such imaging-derived adiposity markers could improve decision-making in equivocal cases, particularly for lesions scored as PI-RADS 3, attributed to more accurate, AI-driven predictive models. These tools could ultimately enhance non-invasive diagnostic strategies and guide personalized management in prostate cancer.

## Figures and Tables

**Table 1:** Demographic data of the patients.

Characteristics	Value
Age, mean ± SD (range)	66.2 ± 11.0 (40–87) years
Age groups, n (%)	<60: 26 (25.0%)
	60–69: 34 (32.7%)
	70–79: 35 (33.7%)
	≥80: 9 (8.7%)
PSA level, median (IQR)	12.2 ng/mL (5.2–23.5)
PI-RADS mpMRI score, n (%)	PI-RADS 1–2: 59 (56.7%)
	PI-RADS 3: 15 (14.4%)
	PI-RADS 4–5: 30 (28.8%)
Prostate volume, mean ± SD	45.8 ± 22.1 mL
Biopsy-naive status	104 (100%)
Periprostatic tissue thickness (cm)	1.13 (1.1–1.15)
ubcutaneous cellular tissue thickness (cm)	3.31 ± 1.18
Periprostatic fat volume (mL)	12.14 ± 3.65
Posterior adipose tissue thickness (cm)	0.76 ± 0.24

**Table 2:** Summary of the main results.

Parameter Analyzed	PI-RADS Correlation (ρ)	*p*-Value	PSA Correlation (ρ)	*p*-Value (PSA)	Notes
Periprostatic fat thickness	−0.142	0.141	−0.162	0.094	No significant correlation overall.
Subcutaneous fat thickness	−0.007	0.931	−0.036	0.718	No correlation detected.
Posterior periprostatic fat thickness	0.240	0.012	–	–	Weak but significant correlation with PI-RADS.
Periprostatic fat volume	0.190	0.059	–	–	Near-significant trend; stronger in younger group.
PI-RADS vs. fat volume (<60 yrs)	0.470	0.0154	–	–	Stronger correlation in younger patients.
PI-RADS vs. posterior fat (≥60 yrs)	0.280	0.013	–	–	Localized fat more relevant in older patients.
PI-RADS vs. subcutaneous fat (≥60 yrs)	−0.226	0.0467	–	–	Weak inverse trend.
Regression model (linear thickness)	R^2^ = 0.019	0.376	–	–	Model not significant; low predictive power.
Group difference (PPAT high vs. low PI-RADS)	Δ = +0.06 cm	0.421	–	–	No statistically significant difference.

## Data Availability

The information is contained within this article in its entirety. For additional information, please feel free to inquire with either the original author or the corresponding author. Public access to data is restricted as a result of the patient privacy standards that regulate the handling of clinical data.
